# The College Library and Museum

**Published:** 1852-01

**Authors:** 


The College Library and Museum.—Those wishing to make donations in books, specimens, &c., &c., to either of these, will find their donations thankfully received and justly appreciated, and also duly credited to the individuals presenting them. We hope soon to have collected a museum which will be a source of attraction to the profession visiting our city.

